# Structure Effects on Swelling Properties of Hydrogels Based on Sodium Alginate and Acrylic Polymers

**DOI:** 10.3390/molecules29091937

**Published:** 2024-04-24

**Authors:** Grzegorz Kowalski, Mariusz Witczak, Łukasz Kuterasiński

**Affiliations:** 1 Department of Engineering and Machinery for Food Industry, Faculty of Food Technology, University of Agriculture in Kraków, ul. Balicka 122, 30-149 Kraków, Poland; m.witczak@urk.edu.pl; 2 Jerzy Haber Institute of Catalysis and Surface Chemistry, Polish Academy of Sciences, ul. Niezapominajek 8, 30-239 Kraków, Poland; lukasz.kuterasinski@ikifp.edu.pl

**Keywords:** hydrogels, poly(acrylic acid), sodium alginate, swelling, DSC, GPC

## Abstract

Hydrogels based on sodium alginate (SA) and partially neutralised poly(acrylic acid) were obtained by radical polymerisation. The hydrogels were cross-linked with N,N′-methylenebisacrylamide (MBA), simultaneously grafting the resulting polymer onto SA. The findings of the FTIR spectroscopy showed that all of the hydrogels were effectively synthesized and sodium alginate was chemically bonded with the poly(sodium acrylate) matrix. DSC analysis of the melting heat and glass transition parameters indicated that the hydrogel structure had changed as a result of the cross-linking process. Sodium alginate and MBA were tested at different concentrations to determine how they affected the hydrogel properties. A very high content of the biopolymer, i.e., sodium alginate, was used in our research, up to 33 wt%. This resulted in durable and stable hydrogels with a very high ability to uptake water, comparable to hydrogels based on synthetic polymers only. The ability to swell is inversely proportional to the quantity of MBA present. By increasing the amount of sodium alginate in the hydrogel, the ability of the hydrogel to absorb water is reduced. However, water uptake remains relatively high at 350 g·g^−1^, even for the hydrogel with the highest SA content.

## 1. Introduction

Hydrogels, which consist of slightly cross-linked hydrophilic polymers, swell rapidly when exposed to water. Polymer backbones, water and cross-linking agents form their three-dimensional network. Hydrogels are versatile absorbents, with applications in fields as varied as agriculture, horticulture, personal care, pharmaceutical delivery and the purification of water by removing metal ions and dye molecules.

Natural polymers are also an alternative for making hydrogels, although synthetic polymers have been cited in a number of research papers [1,2,3]. Due to their biodegradability and biocompatibility, hydrogels derived from natural polymers are preferred for use in food [4], agriculture and pharmaceutical applications [5,6,7,8,9]. Polysaccharides are superior to synthetic polymers because of their numerous useful properties. The synthesis of new hydrogel structures based on synthetic polymers and polysaccharides has been the subject of numerous studies. The most commonly used polysaccharides include chitosan [10,11], alginate [9,12,13,14], starch [15,16,17], cellulose [18], carboxymethylcellulose [19,20], hyaluronic acid [21], etc. These biopolymers have several desirable features, such as being abundant, inexpensive, renewable, biodegradable, polyfunctional, highly reactive chemically, and possessing a wide variety of properties as well as adsorption capacities [15,22,23]. Thus, there has been a surge of recent interest in the method of producing hydrogels from biopolymers.

Sodium alginate (SA) is a water-soluble salt of alginic acid. It is derived from a brown algal polymer. This natural polysaccharide is made up of 1,4-β-d-mannuronic acid blocks and α-l-guluronic acid residues, which contribute to its non-toxic nature. In research by Loureiro Dos Santos, it has been shown that homogeneous blocks of mannuronic and guluronic acid units can interchange within the structure [24]. Alginate occurs naturally in the cell walls and intercellular slime of various brown algae species and certain bacteria, including *Pseudomonas* and nitrogen-fixing bacteria. It is commonly found in the form of a sodium salt [25,26].

Alginate may be gelled and cross-linked by exchanging sodium ions for multivalent cations. Cross-linked hydrogels can be used to deliver bioactive compounds in a controlled manner. It has been demonstrated that hydrogels based on sodium alginate exhibit poor mechanical characteristics [27,28]. Physical or chemical methods can be used to improve the properties of hydrogels based on sodium alginate. Alginate hydrogels are promising because they combine the best qualities of both alginates and hydrogels [29]. Physical cross-linking through the development of ionic bonds between bivalent cations and carboxyl groups on alginate is one of the most common methods for producing alginate-based hydrogels [30]. Despite being inexpensive and easy to use, in research, the resulting gels have been shown to possess poor thermal stability and mechanical properties. As a result, in a growing body of research, alternatives to traditional cross-linking techniques have been observed, such as chemical cross-linking and interpenetrating polymer networks (IPN) [30]. Thus, double cross-linked polymers have been obtained in which SA has been cross-linked with polyvalent cations, while an alternative structure based on acrylic acid (AA) is formed by radical polymerisation in the presence of a cross-linking agent [31] or by polymerisation and cross-linking of AA using microwave radiation [32]. Another approach involves the simultaneous radical polymerisation of the monomer, cross-linking of the polymer and grafting of the resulting polymer onto sodium alginate molecules. In the studies published so far, poly(acrylic acid-acrylamide) copolymers [33], poly(acrylic acid) [12,31,34,35,36,37,38,39] and poly(vinyl alcohol) (PVA) [40] have been used, among others, for this purpose.

Sodium alginate has been used as a matrix in a variety of applications, as made obvious above. Polyacrylic acid derivatives are one of the most commonly used polymers to be grafted onto sodium alginate due to their high ability to absorb water. However, the addition of sodium alginate is relatively low in all studies published to date. It is usually 5–10 wt% relative to the amount of acrylic monomer [11,37,38]. In order to determine the properties of polyacrylic acid and SA based hydrogels at a much higher renewable polymer content, in our study, hydrogels with SA content of up to 33 wt% were prepared. The effect of the amount of cross-linking agent and SA on the swelling properties as well as structure of hydrogels with relatively high SA content has been studied. The obtained hydrogels were subjected to various characterisation techniques. These included analysis of the soluble fraction, swelling behaviour, FTIR, GPC and DSC.

## 2. Results and Discussion

**Figure 1:**
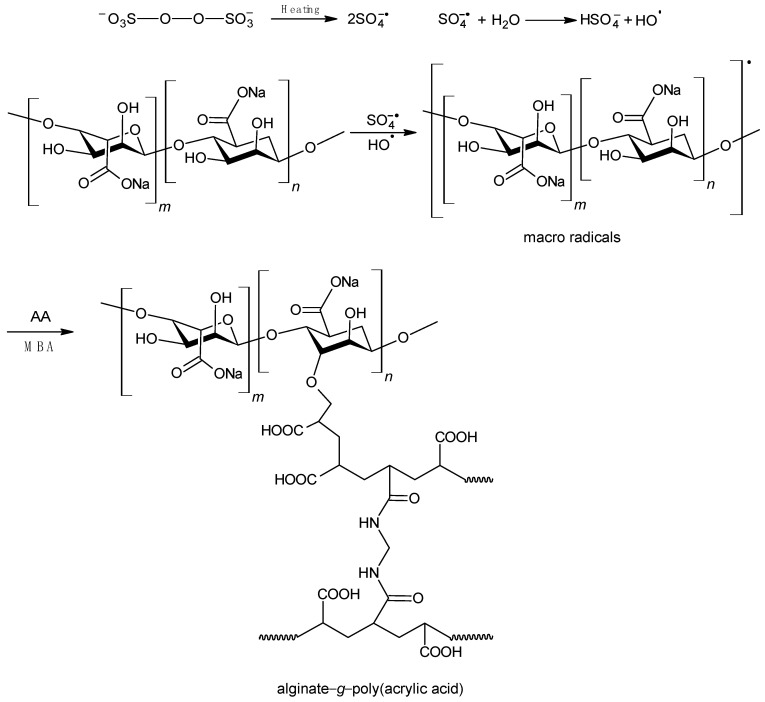
Proposed mechanism for the synthesis of alginate−g−poly(acrylic acid) hydrogels.

**Figure 2:**
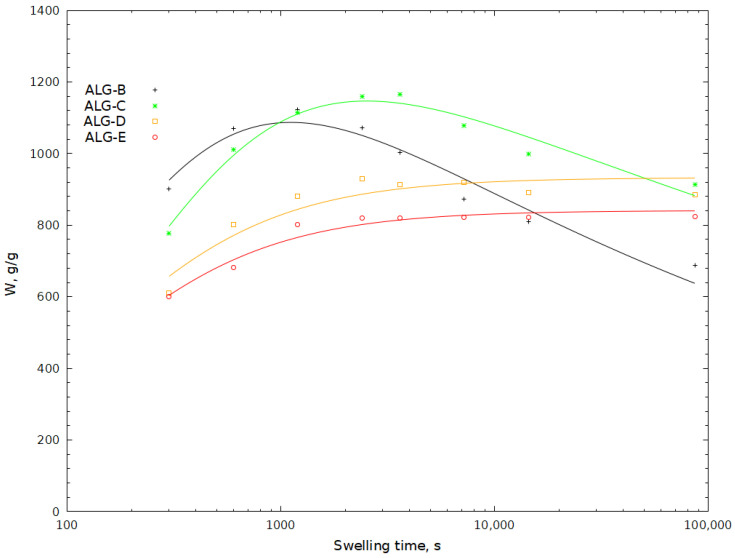
Effect of monomer amount in hydrogel on swelling capacity of hydrogels (ALG-A–ALG-E).

As part of the research presented in this article, hydrogels with excellent swelling ability in aqueous media were synthesised. They were prepared by means of a radical polymerisation reaction between acrylic acid (AA) and sodium acrylate (SA). The resulting polymer chains were simultaneously grafted onto sodium alginate. To create a three-dimensional network, polymerisation was carried out in the presence of a cross-linker, i.e., N,N′-methylenebisacrylamide (MBA). As a result of the process carried out under these conditions, polymers with a cross-linked structure were obtained. The proposed structure and mechanism for the formation of SA-g-poly(acrylic acid) is shown in Figure 1. A similar mechanism has been proposed for hydrogels based on other polysaccharides [21,41]. The reaction to produce the hydrogel materials was conducted under controlled conditions in an inert gas atmosphere. The first batch of hydrogels was synthesised with a variable amount of cross-linker between 0.0149 and 0.1603 g (Table 1) (ALG-AA–ALG-E samples). Another batch of hydrogels was obtained with a fixed amount of MBA at 0.1200 g and a variable amount of sodium alginate between 1–10 g in the reaction mixture (Table 1) (ALG-D1–ALG-D10 samples), after determining the optimum level of cross-linker in the product—sample ALG-D. The optimum cross-linking agent content was determined from observations of hydrogel water uptake (Figure 2) and was defined as the minimum amount of cross-linking agent at which the resulting hydrogel is stable and its water uptake reaches equilibrium over the time period studied. As a result, hydrogels with up to 33 wt% of SA were obtained. Due to the very high viscosity of mixtures containing such a high amount of SA, greater concentrations were not used in the present study. Mixing and equal mutual accessibility regarding all components of the reaction mixture were then hindered, so that the hydrogels obtained could have very uneven structure. The impact of polymerisation reaction parameters on the end-product properties, i.e., swelling capacity and hydrogel swelling kinetics, was studied. The content as well as the molecular weight of the hydrogel soluble fraction was determined using UV-VIS and GPC. Phase transformation parameters were determined via DSC analysis.

### 2.1. Swelling Properties of PAA/SA Hydrogels

To determine the swelling kinetics and capacity of hydrogels, they were immersed in water for defined periods, i.e., 5, 10, 20, 40, 60, 120, 240 and 1440 min. As the size of the hydrogel sample has a significant effect on the diffusion process and the rate of water uptake in the hydrogel, these measurements were carried out on hydrogel with a grain size of 0.12–0.2 mm. The water that was not absorbed by the hydrogel was filtered off, and the swollen gel was weighed. Hydrogels with different structures were produced by modifying the concentration of a cross-linking agent and sodium alginate. The effect of both factors on water absorption capacity was determined. In this group of gels, the molar ratio of MBA in relation to AA ranged from 0.000232 to 0.002498 when the amount of the other reactants was maintained constant (Table 1).

To determine the influence of the cross-linker concentration in the reaction mixture, the swelling of hydrogels in water with a molar ratio of MBA to AA (*x*) ranging from 0.000232 to 0.002498 was studied. No changes were made to any of the other reaction parameters, as shown in Table 1. The findings concerning swelling kinetics of this class of hydrogels are shown in Figure 2. Water absorption characteristics could not be measured for samples ALG-AA and ALG-A; hence, they were not included in Figure 2. These two samples behaved differently from the rest of the batch of hydrogels throughout the experiment. The two hydrogel samples had the lowest amount of cross-linking agent. This concentration of MBA was too low for the formation of a robust and stable network of the hydrogel. These two polymer samples exhibited full solubility in water. After being filtered, no gel residue was left on the sieve. It was found that stable hydrogels could not be produced with such a small amount of MBA. The formed cross-linking bridges were either unstable or there were too few of them for the produced polymer to maintain a stable three-dimensional hydrogel network capable of absorbing and retaining water in its structure. With a molar ratio of MBA to AA (*x*) at 0.000232 and 0.000313, respectively, it seems that the quantity of the cross-linking agent utilised in the production of ALG-AA and ALG-A gels was insufficient to create stable hydrogels. These two samples did not allow for accurate water absorption testing.

The investigated hydrogels demonstrated balanced water uptake values between 820 and 1120 g∙g^−1^. Reports in the literature indicate that when cross-linking agent concentration increases, water absorption decreases [42,43,44]. More of the cross-linking agent results in less water absorption by the hydrogel. This is due to the fact that the mesh size in the gel structure is significantly reduced and the structure of a hydrogel with a high degree of cross-linking is more compact. The water uptake recorded for ALG-B and ALG-E samples decreased as *x* increased. Therefore, the density of the cross-linking in the hydrogel structure was the main factor responsible for the amount of water that the hydrogel was able to absorb. 

Consistent with Flory’s hypothesis and the findings of other researchers [42], the balanced swelling tended to decrease with increasing cross-linking agent concentration.

When analysing the water uptake of individual hydrogels for ALG-B and ALG-C samples, an overshooting effect was observed. This phenomenon is indicated by the fact that the water uptake of the hydrogel reaches a maximal value and then decreases until a state of equilibrium is reached [38,45]. There have been many explanations for the overshooting effect. 

**Figure 3:**
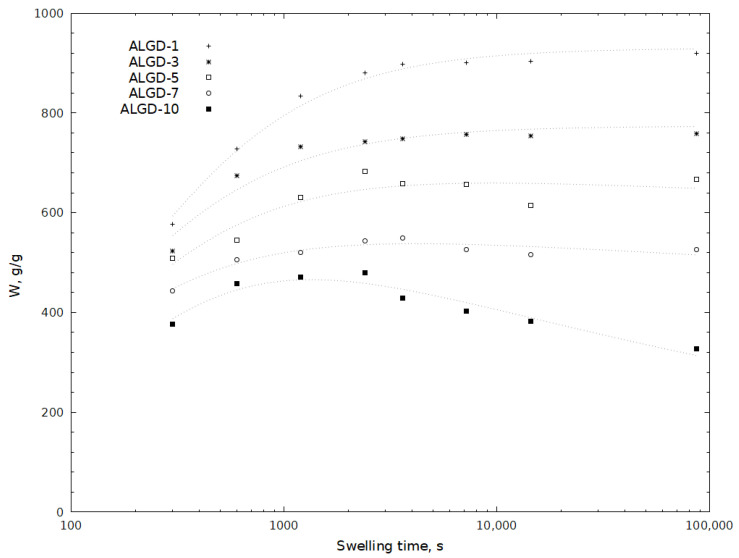
Effect of sodium alginate amount in a hydrogel on the swelling capacity of hydrogels (ALG-D1–ALG-D10).

Peppas et al. proposes that this phenomenon is related to the relaxation process of macromolecular chains. They also indicated that the overshooting effect is greater for loosely cross-linked samples [46,47]. Other authors have attributed the overshooting effect that appears in the swelling curves of poly(N-vinylimidazolium-co-sodium styrene sulfonate) to the formation of additional ionic cross-links [48]. Díez-Peña et al. observed this phenomenon in the case of swelling of cross-linked poly(N-iPAAm-co-MAA) hydrogels. They proposed that the main reason for this phenomenon is the coexistence of chemical and physical cross-linking. This is caused by the formation of hydrogen bonds between the carboxyl and amide groups of the hydrogel in a hydrophobic environment [49]. In the case of relatively lightly cross-linked ALG-B and ALG-C hydrogels, the overshoot phenomenon can be explained by the relaxation of the macromolecular chains [46], which is greater for hydrogels with a lower degree of cross-linking, as shown in Figure 2. We attributed this behaviour to the lower degree of ionisation of the acid, which leads to the formation of hydrogen bonds between the two monomeric units. As the amount of water absorbed increases, the mobility of the polymer chains also increases, allowing the polymer structure to rearrange. The hydrogen bonds thus formed between the monomer molecules would compete with the hydrogen bonding of these units to the water molecules in the solvent, so that the net process is an exchange of hydrogen bonds. A possible reason for the overshooting effect may also be the presence of soluble polymer fractions in the hydrogel, which may be released into solution after swelling of the hydrogel [45]. However, as the hydrogels obtained were washed three times before measurements, it cannot be excluded that some of the polymer chains were still present in the hydrogels. Both of the above described phenomena could potentially occur simultaneously in the hydrogels we studied, on a macro scale causing the effect seen in Figure 2 and Figure 3. The overshooting effect was not observed for the hydrogels with a higher degree of cross-linking, namely ALG-D and ALG-E.

The effect of sodium alginate concentration on hydrogel water uptake was also investigated as part of this study. Gels having the least amount of sodium alginate in their structure are the most stable over time. As the sodium alginate content increases, the changes in the hydrogel configuration over time become greater, demonstrating a decrease in the ability to take in water after 24 h. This phenomenon was most evident for the sample with the highest sodium alginate content—ALGD-10 (Figure 3), in which the water absorption after 24 h was lower than the maximal value for this hydrogel. For samples with lower sodium alginate content, the water uptake after 4 h remained constant. All of the tested hydrogels absorbed water very quickly. The swelling capacity of these hydrogels at equilibrium ranged from 350 to nearly 900 g of water per g of hydrogel, depending on the sodium alginate content. The rate of water absorption in the first five minutes was very high. During this period, hydrogels reached between 60–90% of their maximum water uptake. They achieved their maximal level after 20–40 min (Figure 3). The introduction of a hydrophilic polymer such as sodium alginate into the gel network should increase its ability to uptake water. Furthermore, sodium alginate also provides an electrostatic repulsive force in the network due to its negatively charged carboxylate functional groups (COO^−^ Na^+^). The introduction of an alginate should, therefore, improve the water uptake properties of the hydrogel. Such a phenomenon has been reported by other authors, where an increase in the amount of water absorbed was observed as the SA content of the hydrogel increased to 4 wt%.

Above this value, they observed a decrease in the amount of water that was absorbed [6]. In our study, a similar maximal value was not observed because our hydrogels contained a much higher amount of SA, i.e., from 3.3 to 33 wt%. The swelling capacity of the gels decreased along with increasing alginate concentration. However, their water uptake capacity remained very high. For a hydrogel with 33 wt% of SA, i.e., ALG-D10, the value was still over 300 g/g. Part of the alginate, especially in the case of hydrogels with a high SA content, can fill voids in the network structure, blocking the space that could potentially be available to water molecules. A decrease in the swelling properties of hydrogels with high sodium alginate content may be due to the fact that the alginate fills empty spaces in the hydrogel network that would be available to water molecules. This reduces the possibility of water molecules entering the hydrogel network [3,6].

### 2.2. Analysis of Sol Fraction Content in SA Based Hydrogels

**Figure 4:**
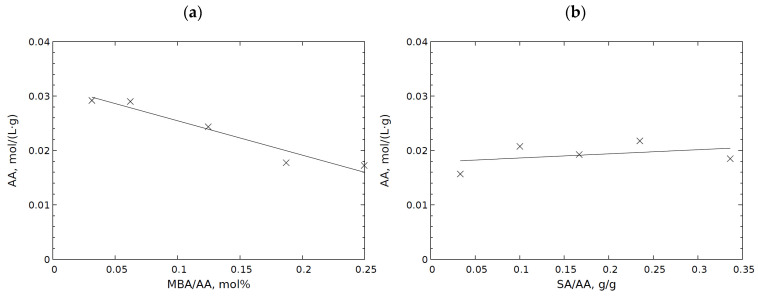
Influence of reaction parameters on content of AA residues in hydrogels for variable amounts of (**a**) MBA (ALG-A–ALG-E), (**b**) sodium alginate (ALG-D1–ALG-D10). The concentration of acrylic acid is related to the weight of the hydrogel.

Due to the possibility of incomplete cross-linking, it was chosen to determine the amount of soluble fractions present in the hydrogels. UV-VIS spectroscopy (band analysis of carbon–carbon double bonds in unreacted acrylic acid) was used to analyse the samples [50]. Some of the produced polymers do not combine to form a polymer network, but rather produce free polymer chains, which accounts for the existence of the soluble fraction. As a result, they are a soluble fraction that is simple to remove. Only trace amounts of acrylic acid residues and soluble short-chain acrylic acid oligomers were detected, both of which may contain C=C double bonds. The probability of SA to react with AA depends on the distance of the acid molecules from the hydroxyl groups of sodium alginate due to the larger and less mobile nature of SA molecules [16,23]. The results of the experiment indicate that higher concentrations of the cross-linking agent have beneficial effects on acrylic acid conversion. Insoluble fractions rich in C=C double bonds were found to somewhat decrease when MBA concentration rose (Figure 4a). It was also shown that this relationship is linear.

In a set of samples with different sodium alginate concentrations (ALG-D1–ALG-D10), practically no effect was observed concerning the amount of sodium alginate on the amount of unreacted AA in the final hydrogel (Figure 4). Despite an increase in the potential amount of hydroxyl groups on sodium alginate, on which the reaction with AA could take place, the expected higher AA over-reactivity was not observed. The grafted polymer chains were best treated with a propagation reaction. In addition, the viscosity of the reaction mixture increased dramatically with increasing sodium alginate content, making it more challenging to stir and interact with the reagents. As a result, these two competing effects neutralised each other, which was subsequently observed on a macro scale as no effect of the amount of sodium alginate on the presence of AA and oligomer fractions.

During the first phase of water absorption, the presence of soluble fractions might slow down the process, although this rate will also be highly dependent on the maximum water absorption capacity of a particular hydrogel. The presence of a solute in the gel causes it to expand, which may be explained by its high chemical potential or the osmotic force it experiences [45].

### 2.3. The FT-IR Spectra of PAA/SA Hydrogels

**Figure 5:**
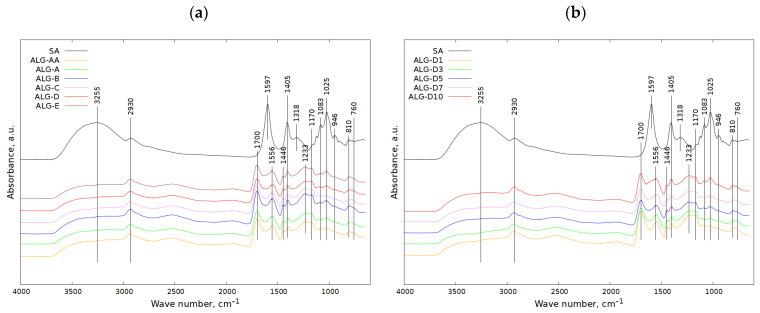
Influence of (**a**) cross-linker and (**b**) SA content on appearance of hydrogel IR spectra.

In Figure 5a, the IR spectrum of pure sodium alginate (line SA) is shown, while the rest of the IR spectra refer to the hydrogels prepared from acrylic acid and sodium alginate with different amounts of a cross-linking agent (MBA). In the considered mixture, the amount of cross-linker gradually changed (from 0.02 to 0.25 mol% with reference to acrylic acid).

For pure sodium alginate (line SA), the band at 3255 cm^−1^ corresponds to the stretching vibration of the OH groups, while the signal at 2930 cm^−1^ can be attributed to the stretching vibrations of the C-H groups. The bands at 1597 cm^−1^ and 1405 cm^−1^ probably come from asymmetrical stretching vibrations of carboxyl. The presence of the band at 1318 cm^−1^ could correspond to bending vibrations of C-H or O-H groups. The bands at 1170–900 cm^−1^ could correspond to C-O-C stretching vibrations or to CH-OH groups [51,52,53,54,55]. The presence of the small signals at 810 cm^−1^ and 760 cm^−1^ could be implied by bending vibrations of carboxylic ions, and rocking vibrations of -CH_2_- groups, respectively [56].

The addition of a cross-linker (MBA) to sodium alginate caused significant changes in the appearance of IR spectra (Figure 5a). The apparent decrease regarding the intensity of the band at 3255 cm^−1^ could take place due to a potential reaction between hydroxyl groups coming from SA and a cross-linker. Therefore, the weakened band at 3255 cm^−1^ can be attributed to either stretching vibrations of OH groups (from acrylic acid, sodium alginate or water) or stretching vibrations of N-H (from cross-linker). Symmetric and asymmetric stretching vibrations of COO- can be assigned to the bands at about 1700 cm^−1^ and 1446 cm^−1^. Both acrylic acid and sodium alginate contain carboxyl ions. Scissoring -CH2- vibrations can also be responsible for the existence of the band at 1446 cm^−1^. The signal at 1556 cm^−1^ can be assigned to amide II. The appearance of the 1402 and 1162 cm^−1^ bands visible for the hydrogels can be further attributed to the -CH2- and OH bending vibrations, respectively, as well as the C=C stretching vibrations of the (C=C)-(C=O) in the cross-linker [56,57,58,59]. The new maximum found at 1233 cm^−1^ can be related to C-O-C groups formed during the reaction between SA and AA in the presence of a cross-linking agent. The weakening of the band at 1405 cm^−1^ and the disappearance of the signals at 1318 cm^−1^ and 946 cm^−1^ are other effects of the reaction between sodium alginate, acrylic acid and cross-linker. The appearance of the IR spectra was not changed by increasing the cross-linker content in the reaction mixture of SA with other components.

The IR spectra of SA and AA hydrogels with varying SA content are shown in Figure 5b. At first sight, the spectra obtained for these hydrogels seemed to be very similar to one another. However, detailed analysis revealed that the intensity of the bands at 1025 cm^−1^ and 1083 cm^−1^ increased as the sodium alginate content in the mixture increased.

Our FT-IR analysis corresponded with the results obtained by Fenoradosoa et al. [51], who characterised alginate extracted from the brown seaweed Sargassum turbinarioides. They indicated the presence of, among others, O-H, C-H, O-C-O, C-O-H, C-C-H, O-C-H and C-O vibrations. Similar results were reported by Pereira et al. [54], who studied thin films based on alginate and aloe vera. 

The results obtained from our FT-IR analysis were in high accordance with the results reported by Helmiyati and M Aprilliza [52], who studied sodium alginate prepared from the extraction of brown algae as an eco-friendly superabsorbent. They also indicated the occurrence of OH groups as well as IR vibrations assigned to C-H groups. 

The findings on structural characterisation performed via the FT-IR technique of our samples also are in line with the structural changes reported by Sinitsya et al. [57], who investigated partially amidated N-alkyl pectin amides, which were prepared by aminodealkoxylation of highly methoxylated pectin with selected primary amines. The amidation with primary amines caused the attachment of various functional groups to a pectin macromolecule, which influenced the physicochemical properties of pectin derivatives. 

According to Qiao et al. [58], who adsorbed water on starch-based superabsorbent polymers prepared in high starch concentrations, apart from C-O-C bands, the occurrence of the bands assigned to C=O, C-N and N-H was shown. They also indicated that the increase in amylopectin reduced the amount of polyacrylamide in superabsorbent polymers but increased the ratio of starch carbons grafted with polyacrylamide. In general, the increases in polyacrylamide in starch and the length of its chain increased water absorption capacity, whereas the denser fractal gels reduced this parameter. In turn, Zhang et al. [59], who synthesised microgels of different cross-linking densities, claimed that sodium trimetaphosphate reacted with the hydroxyl groups of carboxymethyl starch, which led to the generation of ester linkages.

### 2.4. Molecular Weight Distribution of Hydrogel Soluble Fractions

**Figure 6:**
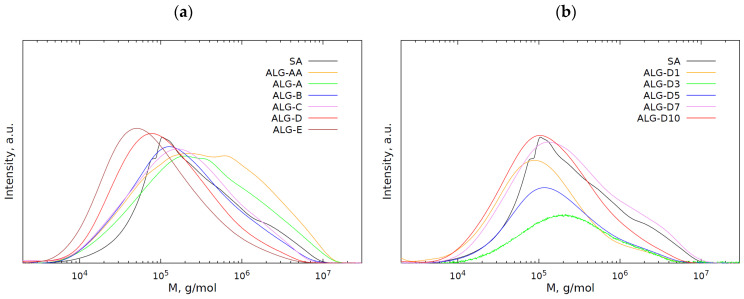
Effects of (**a**) cross-linking agent and (**b**) SA amounts on distribution of molecular weight for SA-based soluble gel fractions.

To accurately analyse the molecular masses of the hydrogel soluble fraction, dried extracts of hydrogel samples were analysed by GPC chromatography (Figure 6). From the analysis of the chromatograms regarding the soluble fractions, the structure of the cross-linked and insoluble hydrogel fractions could be indirectly determined.

The profiles of the molecular weight distribution showed quite large average molecular weights, ranging from 0.41 × 10^5^ to 1.00 × 10^5^ g∙mol^−1^ and from 2.14 × 10^5^ to 1.02 × 10^6^ g∙mol^−1^. The water-soluble fractions had polydispersity ratios between 4.42 and 10.2 (Table 2). These features may be indicative of polymeric compound presence. However, when compared to the average molecular weights of pure sodium alginate, it was clear that the recovered polymers were not sodium alginate in such a form (M_n_ = 1.10 × 10^5^ and M_w_ = 6.49 × 10^5^ g∙mol^−1^), but with a polydispersity ratio (p_d_) of 5.9 (Table 2). Furthermore, it could be observed that as the amount of cross-linking agent (MBA) increased in the ALG-AA–ALG-E samples, the soluble fractions became of increasingly lower average molar mass, which is because the number of polymer chains built into the three-dimensional hydrogel network increased. Their relatively low molecular weights suggest that these polymers do not contain sodium alginate; they could be uncross-linked polymers and poly(acrylic) chains of different lengths. In contrast, for a series of samples in which the amount of sodium alginate (ALG-D1–ALG-D10) was varied at a fixed amount of cross-linking agent, an increase in the number of average molecular weights was observed. These values are close to those of sodium alginate. This behaviour may indicate that some of the sodium alginate is not incorporated into the hydrogel structure and remains in a non-cross-linked form, like poly(acrylic) chains.

### 2.5. Thermal Analysis

**Figure 7:**
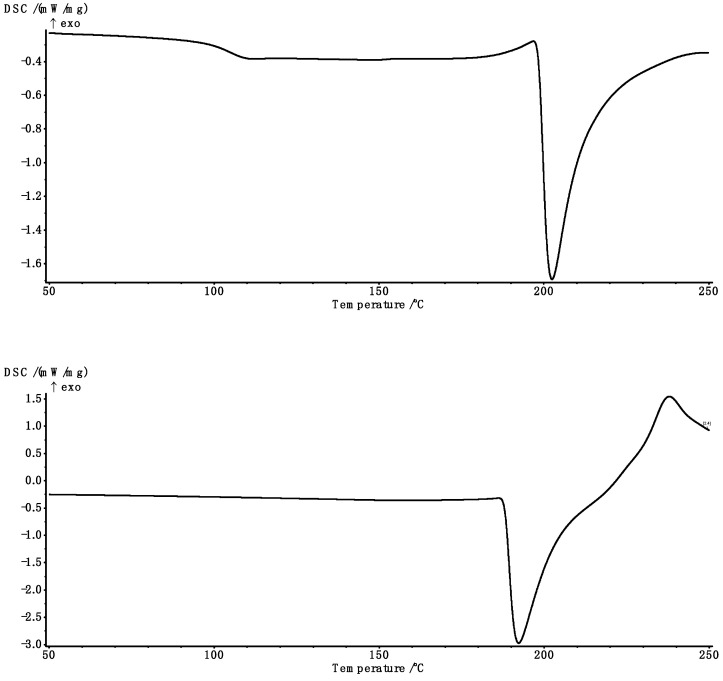
Typical DSC curves of analysed sample (second scan)—5% ALG, 0.120 MBA (**top**) and SA (**bottom**).

In Figure 7, illustrative examples are given of curves obtained using DSC. The cross-linked samples were by a glass transition and a characteristic peak associated with the melting process in the studied range. The results obtained for the parameters characterising both transformations are summarised in Table 3 and Table 4.

**Figure 8:**
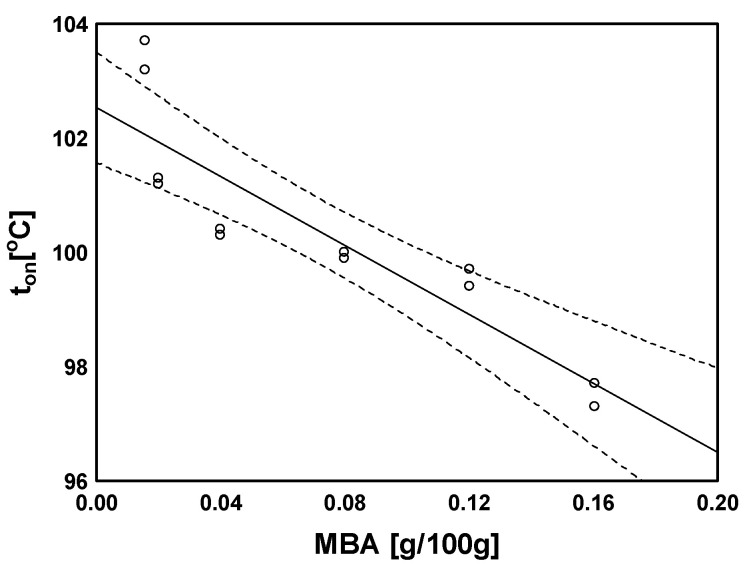
Temperature dependence regarding onset of glass transition on MBA content.

For the control sample, the presence of glass transition was not observed in the analysed range. On the other hand, the maximal glass transition temperature was found for the sample cross-linked with the lowest amount of cross-linking agent (MBA). Increasing the cross-linker content decreased all temperatures characterising the glass transition and increased the transformation range, while there was small variation in specific heat ∆c_p_ (no statistically significant correlation). Statistically significant correlations were found between the temperatures characterising the glass transition and MBA content (r ranging from −0.752 to −0.893 at *p* < 0.005). The strongest correlation characterised the temperature for the onset of the transition (Figure 8). In contrast, for the range of the transition, the correlation was positive (r = 0.786, *p* = 0.002).

**Figure 9:**
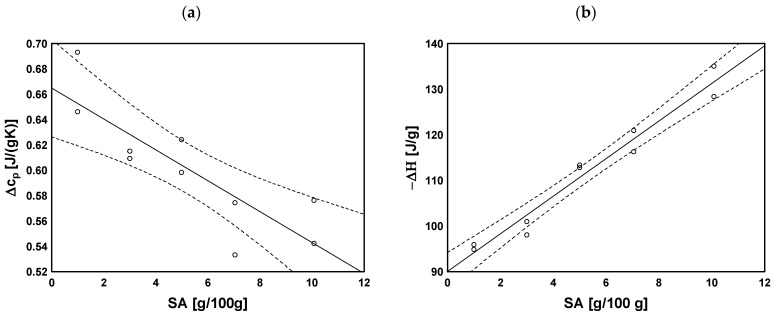
Dependence of (**a**) heat transformation and (**b**) melting heat on SA content.

Similarly to the ALG-AA–ALG-E samples, an increase in SA content (ALG-D1–ALG-D10) resulted in a decrease in transformation characteristic values, with weaker relationships than for samples with variable MBA content, and a statistically significant value was found only for the onset of transformation (r = −0.665, *p* = 0.040). Similarly, a statistically significant but positive correlation coefficient was noted for the transformation temperature range (r = 0.641, *p* = 0.046). However, the strongest correlation with sodium alginate content was found for the heat of transformation (∆c_p_, Figure 9a), which decreased along with increasing SA content (r = −0.844, *p* = 0.002).

It should, therefore, be concluded that the heat of transformation value is determined by the content of SA, whereas in the case of the characteristic transformation parameters, both factors (MBA and SA content) have influence, suggesting that the reaction process and the final value of the degree of cross-linking play a major role.

A strong correlation was found between the proportion of ALG and the enthalpy value (r = 0.980, *p* < 0.001), while for the characteristic temperatures, the correlation was not strong and only statistically significant for the end of the transformation (Figure 9b). Uncross-linked sodium alginate showed the highest ∆H and lowest temperatures of transformation. For hydrogels with different amounts of SA and MBA, a decrease in the analysed values was observed, followed by an increase, both as the amounts of the cross-linking agent and SA were increased. This indicates an increasing degree of cross-linking in both cases. With regard to MBA, the effect was small and not statistically significant, although with a significant tendency.

Taking the change in peak alignment as well as the values and characteristics of the curve into account, it should be concluded that a cross-linking reaction had taken place. The increase in melting heat indicated an increasing degree of cross-linking and crystallinity of the analysed materials.

## 3. Materials and Methods

### 3.1. Materials

In this work, sodium alginate (SA, M_w_ = 110,000 g mol^−1^, p_d_ = 5.9) purchased from Regis (Bochnia, Poland) was used. Acrylic acid (AA) and N,N′-methylenebisacrylamide (MBA) were procured from Fluka (Buchs, Switzerland). Potassium persulfate and NaOH were purchased from POCH (Gliwice, Poland). All reagents were of analytical grade.

### 3.2. Synthesis of Alginate-Based Hydrogels

Sodium alginate-based hydrogels were prepared by the polymerisation reaction of acrylic acid, partially neutralised with NaOH to sodium acrylate, with simultaneous grafting of the resulting polymer onto the sodium alginate. N, N′-methylenebisacrylamide was used as the cross-linking agent. The reaction was carried out at 80 °C, in an inert gas atmosphere according to a previously described procedure [16,19]. After cooling, reaction mixtures were washed 3 times with a large amount of 80:20 methanol:water solution. The obtained hydrogels were dried, ground, finally dried at 60 °C for 48 h and separated into fractions of different grain sizes by means of sieves.

### 3.3. Determination of Unreacted Fraction

In a flask containing 100 mL of 1.0% aqueous sodium chloride solution, 0.1 g of the hydrogel was added. The suspension was allowed to shake for 72 h. The insoluble hydrogel was filtered off, and then the obtained solution was analysed. The analysis was carried out in a UV-VIS spectrophotometer (Labomed, Inc., Los Angeles, CA, USA) in a range between 180 and 300 nm. For the hydrogels that were analysed, the absorption maxima were observed at 208 nm. Fixed concentrations of acrylic acid solutions were utilized to calibrate the spectrophotometer.

### 3.4. GPC Measurements of Soluble Hydrogel Fractions

For further investigation, the molecular weight distribution concerning the soluble fractions of the hydrogel was analysed. These were isolated by placing the hydrogel in distilled water for 24 h, after which the soluble fraction was decanted. This procedure was repeated three times. The water was then evaporated, and the extracts thoroughly dried at 45 °C. The dried aqueous extracts were subjected to GPC analysis. The concentration of the samples used for analysis was 5 mg/mL. GPC analysis was performed at 25 °C with an eluent flow rate of 0.6 mL/min. A three-column system consisting of an Ultrahydrogel 2000 (Waters, Milford, MA, USA), Ultrahydrogel 500 (Waters) and Ultrahydrogel 120 (Knauer, Germany) was connected to an RI detector (Knauer, Berlin, Germany). A 0.1 mol/L aqueous solution of NaNO_3_ with the addition of sodium azide was used as the eluent. Pullulan standards (Shodex, Yokohama, Japan) were used to calibrate the instrument.

### 3.5. FT-IR Spectroscopy

FT-IR measurements were performed using the Nicolet 6700 spectrometer with an MCT detector and ATR adapter (Thermo Scientific, Waltham, MA, USA). The IR measuring range was 650–4000 cm^−1^. For each spectrum, 128 scans were taken. The FT-IR spectra are shown in Figure 5.

### 3.6. Swelling Properties

Approximately 0.2 g of the hydrogel samples with a grain size of 0.12–0.2 mm was placed in 500 mL distilled water at 25 °C for a specified time. During this time, the suspension was gently stirred. Subsequently, the unbound water was quickly removed by filtering through a stainless-steel sieve with a mesh size of 0.5 mm. The samples were weighed. Measurements were repeated three times for each sample. The swelling factor was calculated from Equation (1):

(1) $$W=\frac{M_{w}-M_{d}}{M_{d}}$$

where:

W—swelling ratio at time t; 

M_w_—weight of swollen gel at time t [g];

M_d_—weight of dry gel [g].

### 3.7. Thermal Characterisation

Differential scanning calorimetry (DSC) using the DSC 204F1 Phoenix instrument (Netzsch, Selb, Germany) was used to determine the thermodynamic properties of the analysed samples (approx. 15 mg), which were placed in hermetically sealed aluminium vessels and heated to 150 °C, then cooled down to 0 °C and finally heated to 250 °C. The Proteus Analysis software v.8.12 (Netzsch, Selb, Germany) was used to determine characteristic parameters, i.e., transition temperature onset (T_onsetg_), medium (T_midg_), inflection (T_infg_), end-set (T_endg_), glass transition temperature and onset (T_onsetm_), peak (T_pm_), and end-set (T_endm_), as well as the change in heat capacity (Δc_p_) and melting heat (ΔH). The glass transition temperature was assumed as the centre temperature of the T_midg_ transformation, hereafter referred to as T_g_. The tests were performed twice.

### 3.8. Statistical Analyses

One-factor analysis of variance and the least significant difference (LSD) calculated using Fisher’s test at a 0.05 significance level were used to determine statistical differences between means. Pearson’s correlation coefficient values were applied to identify relationships between the parameters. All calculations were performed with Statistica 12.0 (StatSoft Inc., Tulsa, OK, USA).

## 4. Conclusions

Sodium alginate-based hydrogels were prepared by means of radical polymerisation of acrylic acid, sodium acrylate and sodium alginate. MBA was used to cross-link the hydrogels. Hydrogels with a very high content of the renewable polymer, i.e., sodium alginate, were obtained, both of which were stable and had high water uptake.

For all of the gels, the content of the soluble fraction was determined, and FTIR, GPC and DSC studies were conducted. FTIR and GPC studies allowed us to confirm that the alginate-polyacrylate network was formed as a three-dimensional cross-linked hydrogel structure, formed by simultaneous polymerisation and cross-linking reactions. It should be noted that sodium alginate was incorporated into the hydrogel structure, as analysis of the soluble fractions by GPC indicated that sodium alginate was not found in the soluble fractions. DSC analysis of the melting heat and glass transition parameters indicated that the hydrogel structure had changed because of the cross-linking process. The swelling capacity and swelling parameters of the hydrogel network in an aqueous medium were also evaluated. Extremely high water absorption capacity was observed in the synthesised alginate-polyacrylic hydrogels, with certain hydrogels capable of absorbing up to 1000 g of water per 1 g of the hydrogel. However, in the case of loosely cross-linked hydrogels, a relaxation of the macromolecular chains could be observed, which was noted on a macro scale as an overshooting effect. Increasing the content of the cross-linking agent reduced water uptake. The ability to absorb water decreased with increasing sodium alginate content. However, even with 33 wt% of SA, 1 g of hydrogel was able to absorb up to 350 g of water. 

The present research not only provides valuable information on the structure and swelling characteristics of PAA/SA hydrogels, but also provides a base for additional research examining other hydrogels based on acrylic polymers and polysaccharides. With relatively high renewable polymer (SA) content, our study showed that hydrogels with water uptake comparable to synthetic-polymer-only hydrogels can be obtained. Given the high content of renewable and biodegradable polymers in the obtained hydrogels, it seems reasonable to carry out further research on the possibility of using this type of polymer as a matrix for the slow release of fertilisers or metal ion-absorbing agents in aqueous solutions. As increasing the sodium alginate content results in a decrease in swelling properties, it would be necessary to optimise the polymerisation process (e.g., temperature, concentration, amount of initiator, changing the cross-linking agent) so that the desired swelling properties could be achieved with the highest possible amount of renewable polymer.

## Figures and Tables

**Table 1:** Summary of reagents used for preparation of PAA/SA hydrogels.

Sample	SA [g]	AA [g]	MBA [g]	K_2_S_2_O_8_ [g]	NaOH [g]
ALG-AA	5	30	0.0149	0.2	5
ALG-A	5	30	0.0201	0.2	5
ALG-B	5	30	0.0399	0.2	5
ALG-C	5	30	0.0800	0.2	5
ALG-D	5	30	0.1201	0.2	5
ALG-E	5	30	0.1603	0.2	5
ALG-D1	1	30	0.1200	0.2	5
ALG-D3	3	30	0.1200	0.2	5
ALG-D5	5	30	0.1200	0.2	5
ALG-D7	7	30	0.1200	0.2	5
ALG-D10	10	30	0.1200	0.2	5

The amount of water for the preparation of each solution: SA—100 mL, AA—10 mL, MBA—13 mL, K_2_S_2_O_8_—13 mL, NaOH—15 mL. Additional water used in the reaction, e.g., flushing—50 mL.

**Table 2:** Summary of calculated molecular weights regarding soluble fractions of PAA/SA hydrogels.

Sample	M_n_ × 10^−5^ [g∙mol^−1^]	M_w_ × 10^−5^ [g∙mol^−1^]	p_d_
SA	1.10	6.49	5.9
ALG-AA	1.00	10.20	10.2
ALG-A	0.98	9.02	9.2
ALG-B	0.77	4.86	6.3
ALG-C	0.51	3.29	6.5
ALG-D	0.53	3.30	6.2
ALG-E	0.41	2.14	5.2
ALG-D1	0.55	2.64	4.8
ALG-D3	0.89	3.91	4.4
ALG-D5	0.85	3.83	4.5
ALG-D7	0.94	4.49	4.8
ALG-D10	0.97	3.49	5.2

**Table 3:** Parameters characterising glass transition process.

Sample	ALG [g]	MBA [g]	T_on_ [°C]	T_mid_ [°C]	T_inf_ [°C]	T_end_ [°C]	T_end_–T_on_ [°C]	Δc_p_ [J·g^−1^·K^−1^]
SA	-	-	nd	nd	nd	nd	nd	nd
ALG-AA	5	0.016	103.5 ± 0.35 ^h^	109.7 ± 0.28 ^i^	111.9 ± 0.14 ^f^	115.6 ± 0.07 ^g^	12.1 ± 0.28 ^ab^	0.593 ± 0.0240 ^abc^
ALG-A	5	0.020	101.3 ± 0.07 ^g^	107.5 ± 0.07 ^h^	109.6 ± 0.14 ^e^	113.1 ± 0.14 ^f^	11.9 ± 0.21 ^ab^	0.592 ± 0.0156 ^abc^
ALG-B	5	0.040	100.4 ± 0.07 ^f^	106.7 ± 0.07 ^g^	109.3 ± 0.00 ^e^	112.6 ± 0.14 ^ef^	12.3 ± 0.21 ^bc^	0.633 ± 0.0042 ^cd^
ALG-C	5	0.080	100.0 ± 0.07 ^ef^	106.8 ± 0.07 ^g^	109.5 ± 0.21 ^e^	113.3 ± 0.07 ^f^	13.3 ± 0.14 ^cd^	0.628 ± 0.0417 ^cd^
ALG-D	5	0.120	99.6 ± 0.21 ^e^	106.0 ± 0.35 ^f^	108.1 ± 0.07 ^d^	111.9 ± 0.21 ^de^	12.3 ± 0.00 ^bc^	0.611 ± 0.0184 ^abc^
ALG-E	5	0.160	97.5 ± 0.28 ^c^	104.9 ± 0.28 ^e^	107.8 ± 0.35 ^d^	111.6 ± 0.42 ^d^	14.1 ± 0.14 ^d^	0.592 ± 0.0106 ^abc^
ALG-D1	1	0.120	97.2 ± 0.21 ^bc^	102.9 ± 0.07 ^b^	105.3 ± 0.07 ^b^	108.3 ± 0.14 ^b^	11.2 ± 0.35 ^a^	0.670 ± 0.0332 ^d^
ALG-D3	3	0.120	98.0 ± 0.14 ^d^	104.1 ± 0.07 ^d^	106.7 ± 0.14 ^c^	109.9 ± 0.21 ^c^	11.9 ± 0.35 ^ab^	0.612 ± 0.0042 ^bc^
ALG-D5	5	0.120	99.6 ± 0.21 ^e^	106.0 ± 0.35 ^f^	108.1 ± 0.07 ^d^	111.9 ± 0.21 ^de^	12.3 ± 0.00 ^bc^	0.611 ± 0.0184 ^abc^
ALG-D7	7	0.120	97.0 ± 0.28 ^b^	103.5 ± 0.49 ^c^	104.6 ± 1.34 ^b^	109.3 ± 0.92 ^c^	12.3 ± 1.20 ^bc^	0.554 ± 0.0290 ^a^
ALG-D10	10	0.120	93.4 ± 0.21 ^a^	99.7 ± 0.35 ^a^	102.5 ± 0.21 ^a^	105.8 ± 0.35 ^a^	12.4 ± 0.14 ^bc^	0.559 ± 0.0240 ^ab^
One-way ANOVA—*p*			<0.001	<0.001	<0.001	<0.001	0.003	0.016

Differences between values indicated by the same letters in particular columns are non-significant at the 0.05 level of confidence; nd—not detected. Abbreviations: T_on_—onset of glass transition, T_mid_—midpoint of glass transition, T_inf_—inflection of glass transition, T_end_—endpoint of glass transition, Δc_p_—change in heat capacity.

**Table 4:** Parameters characterising melting process.

Sample	ALG [g]	MBA [g]	T_on_ [°C]	T_p_ [°C]	T_end_ [°C]	T_end—_T_on_ [°C]	ΔH [J·g^−1^]
SA	-	0	187.8 ± 0.49 ^a^	192.1 ± 0.35 ^a^	203.0 ± 0.21 ^a^	15.2 ± 0.71	179.4 ± 8.06 ^e^
ALG-AA	5	0.016	202.0 ± 0.14 ^bcd^	205.4 ± 0.07 ^bcde^	213.6 ± 0.21 ^bc^	11.6 ± 0.35	108.4 ± 8.91 ^abc^
ALG-A	5	0.020	202.2 ± 2.69 ^cd^	205.7 ± 2.90 ^cde^	214.8 ± 4.17 ^bc^	12.6 ± 1.48	108.3 ± 9.40 ^abc^
ALG-B	5	0.040	205.2 ± 0.21 ^d^	208.8 ± 0.21 ^e^	217.8 ± 0.35 ^c^	12.6 ± 0.14	108.8 ± 3.75 ^bc^
ALG-C	5	0.080	203.7 ± 0.49 ^d^	207.1 ± 0.07 ^de^	215.2 ± 1.48 ^bc^	11.5 ± 1.98	113.3 ± 6.36 ^c^
ALG-D	5	0.120	200.4 ± 3.04 ^bcd^	204.4 ± 2.62 ^bcde^	215.7 ± 0.49 ^bc^	15.3 ± 2.55	113.0 ± 0.42 ^c^
ALG-E	5	0.160	201.2 ± 3.68 ^bcd^	204.7 ± 3.75 ^bcde^	213.1 ± 4.03 ^bc^	11.9 ± 0.35	113.1 ± 3.25 ^c^
ALG-D1	1	0.120	203.9 ± 0.28 ^d^	207.3 ± 0.07 ^de^	215.9 ± 0.49 ^bc^	12.0 ± 0.78	95.3 ± 0.71 ^a^
ALG-D3	3	0.120	196.8 ± 5.23 ^bc^	201.3 ± 4.31 ^bc^	213.1 ± 0.85 ^bc^	16.3 ± 4.38	99.4 ± 2.08 ^ab^
ALG-D5	5	0.120	200.4 ± 3.04 ^bcd^	204.4 ± 2.62 ^bcde^	215.7 ± 0.49 ^bc^	15.3 ± 2.55	113.0 ± 0.42 ^c^
ALG-D7	7	0.120	199.7 ± 0.21 ^bcd^	203.4 ± 0.42 ^bcd^	213.7 ± 0.71 ^bc^	14.1 ± 0.49	118.6 ± 3.32 ^c^
ALG-D10	10	0.120	196.4 ± 0.99 ^b^	200.5 ± 0.35 ^b^	211.7 ± 1.48 ^b^	15.3 ± 2.47	131.6 ± 4.67 ^d^
One-way ANOVA—*p*			0.001	<0.001	0.001	0.180	<0.001

Differences between values indicated by the same letters in particular columns are non-significant at the 0.05 level of confidence. Abbreviations: T_on_—onset of melting, T_p_—peak of melting, T_end_—endpoint of melting, ΔH—melting heat.

## Data Availability

Additional data will be available upon reasonable request.
